# Translocation of signalling proteins to the plasma membrane revealed by a new bioluminescent procedure

**DOI:** 10.1186/1471-2121-12-27

**Published:** 2011-06-09

**Authors:** Carlotta Giorgi, Anna Romagnoli, Chiara Agnoletto, Leda Bergamelli, Giovanni Sorrentino, Marisa Brini, Tullio Pozzan, Jacopo Meldolesi, Paolo Pinton, Rosario Rizzuto

**Affiliations:** 1 Department of Experimental and Diagnostic Medicine, Section of General Pathology, Interdisciplinary Center for the Study of Inflammation (ICSI) and LTTA center, University of Ferrara, Ferrara; Italy; 2 Aequotech s.r.l., Ferrara, Italy; 3 San Raffaele Scientific Institute and IIT Network, Milan, Italy; 4 Dept. Biochemistry and Dept. Experimental Veterinary Sciences, University of Padua, Padua, Italy; 5 Dept. Biomedical Sciences, University of Padua, and CNR Institute of Neuroscience, Padua Unit, Italy; 6 Venetian Institute of Molecular Medicine, Padua, Italy

## Abstract

**Background:**

Activation by extracellular ligands of G protein-coupled (GPCRs) and tyrosine kinase receptors (RTKs), results in the generation of second messengers that in turn control specific cell functions. Further, modulation/amplification or inhibition of the initial signalling events, depend on the recruitment onto the plasma membrane of soluble protein effectors.

High throughput methodologies to monitor quantitatively second messenger production, have been developed over the last years and are largely used to screen chemical libraries for drug development. On the contrary, no such high throughput methods are yet available for the other aspect of GPCRs regulation, i.e. protein translocation to the plasma membrane, despite the enormous interest of this phenomenon for the modulation of receptor downstream functions. Indeed, to date, the experimental procedures available are either inadequate or complex and expensive.

**Results:**

Here we describe the development of a novel conceptual approach to the study of cytosolic proteins translocation to the inner surface of the plasma membrane. The basis of the technique consists in: i) generating chimeras between the protein of interests and the calcium (Ca^2+^)-sensitive, luminescent photo-protein, aequorin and ii) taking advantage of the large Ca^2+^ concentration [Ca^2+^] difference between bulk cytosolic and the sub-plasma membrane rim.

**Conclusion:**

This approach, that keeps unaffected the translocation properties of the signalling protein, can in principle be applied to any protein that, upon activation, moves from the cytosol to the plasma membrane.

Thus, not only the modulation of GPCRs and RTKs can be investigated in this way, but that of all other proteins that can be recruited to the plasma membrane also independently of receptor activation.

Moreover, its automated version, which can provide information about the kinetics and concentration-dependence of the process, is also applicable to high throughput screening of drugs affecting the translocation process.

## Background

Translocation of proteins, from the bulk of the cytosol to the plasma membrane, is a critical step in the transfer of information from membrane-embedded receptors to the cell interior. Just to cite a few examples, both RTKs and GPCRs recruit, upon activation, effector proteins such as SH2-containing adaptor proteins [1], various enzymes and the desensitizing proteins arrestins [2]. In addition, other classes of effector proteins, such as those of the broad protein kinase C family [3], are activated upon translocation to the plasma-membrane microenvironment, independently of receptor binding. Detailed knowledge of the translocation of specific proteins, not only in physiological, but also in pathological conditions, may therefore highlight key aspects of defined signalling pathways. Impaired or excessive translocations of specific proteins are known in fact to play important roles in the pathogenesis of diseases (see for example the case of the beta 2 isoform of protein kinase C, PKCβII, in hyperglycemia and vascular complications of diabetes mellitus [4,5]).

In the past, the classical approaches employed to investigate translocation were immunocytochemistry and subcellular fractionation followed by western blotting of the isolated fractions. Although adequate to reveal the existence of the process these approaches suffered of many drawbacks and limitations: possible artefacts, low time and space resolution, low sensitivity, problems in the precise identification of the cellular structures involved, that have limited their use to the low-throughput investigation.

The introduction of GFP fluorescent probes and, more recently, of the restoration of the β-galactosidase activity following complementation of two inactive fragments of the enzyme [6], has made possible the kinetics of protein translocation to be investigated in living cells. However, with GFP probes the increases in fluorescence are relatively small, often mixed up by the interference of endogenous fluorophores. The novel β-galactosidase complementation procedure is intrinsically complex. Two chimeric reporters, the translocating protein and its specific target, localized in a precise subcellular structure, are necessary. The translocation process is inevitably influenced by the binding of the two fragments and the proteins addressed to unknown targets cannot be investigated, limiting this technique for a screening purpose.

**Figure 1:**
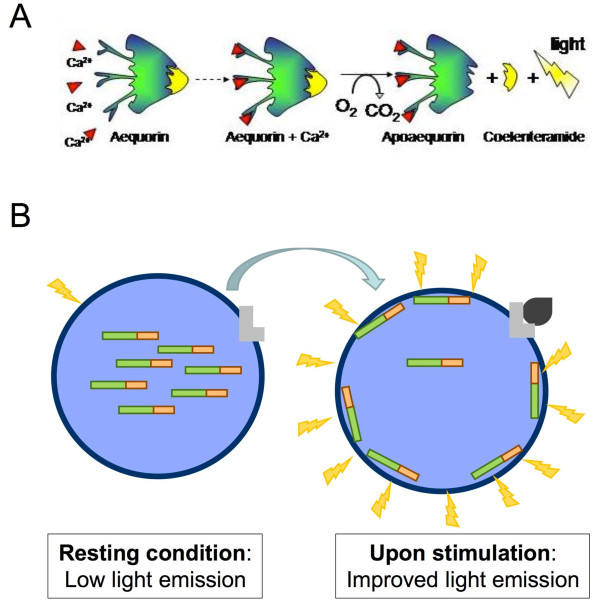
**Schematic representation of the concept behind the new technology**. **A**. The Ca^2+^-sensitive photoprotein aequorin in the active form. includes an apoprotein and a covalently bound prosthetic group (coelenterazine). When Ca^2+ ^ions bind to three high-affinity sites (EF-hand type), aequorin undergoes an irreversible reaction, in which a photon is emitted. **B**. Translocation studies can be performed on aequorin-tagged proteins, thanks to the different [Ca^2+^] present in distinct subcellular compartments. An aequorin-tagged signalling protein (proteinX-Aeq, green/orange sticks) located in the cytosol (light blue) translocates, upon stimulation in a receptor dependent or independent manner, to the plasma membrane (dark blue), where [Ca^2+^] is at least 1 order of magnitude higher then the cytosol, giving rise to an easily detectable increase in light emission (yellow flashes).

Here we have employed a new procedure based on the use of chimeric constructs where the translocation protein is included in frame with aequorin, a well known bioluminescent photoprotein, *per se *a Ca^2+ ^probe (Figure 1A), used so far primarily for the study of Ca^2+ ^homeostasis [7]. The translocation of the aequorin-containing chimera to the sub-plasmalemma rim is able in itself to detect such signal because of the much higher [Ca^2+^] (at least 1 order of magnitude with respect to the rest of the cytosol [8]) generated locally upon cell stimulation (Figure 1B). Given the strength of the light signal emitted by aequorin upon Ca^2+ ^binding (proportional to almost the 3rd power of the [Ca^2+^] [7]), and the lack of bioluminescent molecules within mammalian cells, the procedure has an excellent signal-to-noise ratio.

Here we report results obtained by following the translocation of two interesting proteins (Figure 1C): β-arrestin-2 (βarr2), addressed to a precise receptor target; and PKCβII, for which no protein target is known in the plasma membrane. By the use of a robot the procedure could be made more powerful, providing information about specific aspects of the translocation process, including its time-course and the dose-dependence of its activation.

## Results

### Generation of chimeric probes

Cohesive cDNA constructs, encoding aequorin and β-arrestin-2 or PKCβ were fused in frame and the reporter chimeric proteins are denominated βarr2-AEQ and PKCβ-AEQ (Figure 1C), respectively.

**Figure 2:**
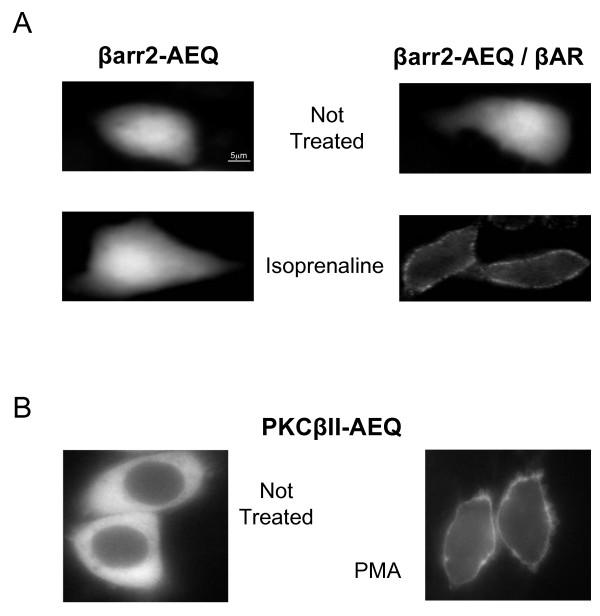
**Confocal microscopy analyses of probes translocation**. **A**. Immunocytochemistry analyses of βarr2-AEQ localization in HeLa cells, either co-transfected with β_2_AR (right panels) or with an empty vector (left panels), in control condition (upper panels) and upon 40 μM isoprenaline stimulation (1 min) in KRB/EGTA (lower panels). After 1 minute of isoprenaline stimulation, the typical re-localization at the plasma membrane, in almost 80% of the cells, was observed only for βarr2-AEQ co-expressed with β_2_AR (lower right panel) because the expression of the endogenous receptors targets of βarr2 is very low in these cells. **B**. PKCβII-AEQ localization in HeLa cells before (cytosolic, left panel) and after 5 minutes of 1 μM PMA treatment in KRB/EGTA (plasma membrane, right panel). After PMA stimulation almost all (≥ 95%) of the cells showed a PKCβII-AEQ plasma membrane translocation.

The initial experiments were carried out to establish, using the classical approaches, i.e. immunocytochemistry (Figure 2) and subcellular fractionation followed by western blot of the fractions (not shown), whether the aequorin tag does affect or not the activity-dependent translocation of the two proteins investigated, βarr2 and PKCβII, after agonists or pharmacological stimulations. The cells used for the studies were HeLa, that do express only very low levels of endogenous β_-_adrenergic receptor (βAR). The βarr2-AEQ cDNA was therefore co-transfected transiently with either the β_2_AR [9] or an empty vector, and the distribution of βarr2-AEQ probe was established before and after stimulation with the receptor agonist, isoprenaline (40 μM, Figure 2A).

In resting cells βarr2-AEQ exhibited a diffuse cytoplasmic distribution (Figure 2A, upper images); upon isoprenaline addition the chimera was rapidly (within 1 min) translocated to the periphery, however only in the cells transfected with the β_2_AR (see the bottom right image of Figure 2A). The stimulation time chosen for the luminescence assays with this chimera was therefore of 1 min.

Other batches of HeLa cells were transfected with the PKCβII-AEQ cDNA and treated with phorbol 12-myristate 13-acetate (PMA), a potent activator of classical PKCs [10]. In this case, the translocation of the probe, from its diffuse distribution at rest to a peripheral distribution, required less than 5 min of PMA treatment (1 μM, Figure 2B).

These times match those reported previously for the translocations of the two proteins upon the two types of stimulation. Thus, the aequorin tag does not affect the activity-dependent translocation of either investigated protein.

### Analysis of βarr2-AEQ and PKC-AEQ translocation by luminescence assay

**Figure 3:**
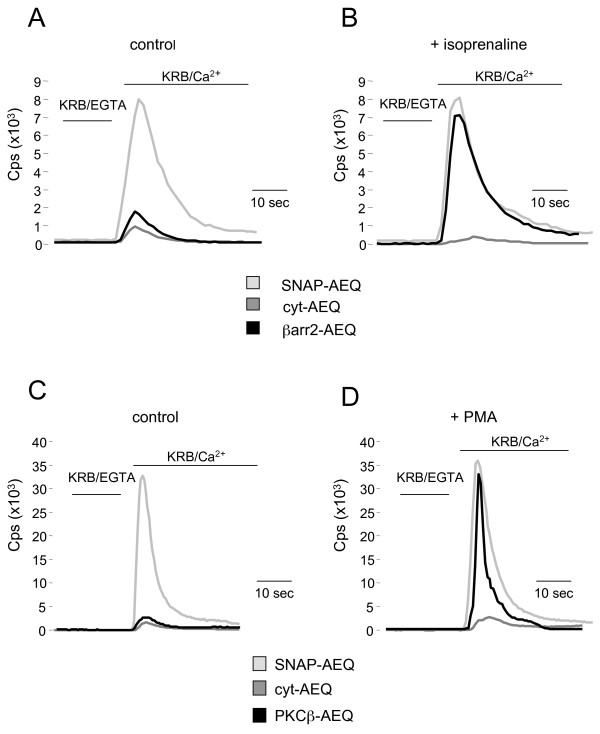
**Assessing the system functioning**. **A-B**. HeLa cells expressing βAR and transfected with βarr2-AEQ or with control probes cyt-AEQ and SNAP-AEQ were perfused with a Ca^2+^-free buffer (KRB/EGTA), showing light emission values slightly above the background (< 100 cps). In control conditions **(A) **upon Ca^2+ ^re-addition to the medium (KRB/Ca^2+^) the light emission values changed markedly only for SNAP-AEQ probe, as expected; while upon isoprenaline stimulation **(B) **also the βarr2-AEQ probe gave rise to a similar light emission increase, confirming probe translocation (βarr2-AEQ control peak 1,730 ± 356 cps n = 9, βarr2-AEQ + isoprenaline peak 6,951 ± 797 cps n = 9; cyt-AEQ control peak 756 ± 36 cps n = 9, cyt-AEQ + isoprenaline peak 562 ± 37 cps n = 9; SNAP-AEQ control peak 8,040 ± 353 cps n = 9, SNAP-AEQ + isoprenaline peak 7,930 ± 80 cps n = 9). **C-D**. A similar behaviour was observed in HeLa cells over-expressing the PKC βII-AEQ probe. Only upon PMA stimulation **(D) **an increase in light emission after Ca^2+ ^re-addition was observed, confirming the efficacy of the assay (PKCβII-AEQ control peak 4,115 ± 1,041 cps n = 12, PKCβII-AEQ + PMA 44,140 ± 7,858 cps n = 15; cyt-AEQ control peak 3,884 ± 785 cps n = 10, cyt-AEQ + PMA peak 3,501 ± 519 cps n = 9; SNAP-AEQ control peak 42,517 ± 5,012 cps n = 12, SNAP-AEQ + PMA peak 44,782 ± 5,749 cps n = 11). All results represent cell populations measurements and are expressed as mean ± standard error (SE). The traces showed correspond to a sample representative of the mean obtained from all experiments. n = number of samples (wells) analyzed from at least ten independent experiments.

To confirm the validity of the aequorin chemiluminescence procedure to reveal the differential distribution of a protein in the subplasmalemma rim and the cytosol, HeLa cells were seeded onto 13 mm coverslips and transiently transfected with two chimeric aequorin cDNAs, one encoding a constitutively cytosolic probe (cyt-AEQ) [11], the other a probe addressed to the cytosolic face of the plasma membrane (SNAP-AEQ) [8]. Thirty six hr after transfection, the probes were reconstituted adding 5 mM coelenterazine in Krebs-Ringer modified Buffer (KRB) and the coverslips were transferred to the chamber of a single-well luminometer. With both the cyt-AEQ and SNAP-AEQ probes the traces recorded in Ca^2+^-free buffer (KRB/EGTA) were slightly above background (< 100 cps) (Figure 3). Upon re-addition of 1 mM CaCl_2 _(KRB/Ca^2+^), the rapid Ca^2+ ^influx (induced by the store depletion occurred during Ca^2+^-free incubation) caused an increase in light emission. As expected, such an increase was over 10-fold greater in the cells expressing the SNAP-AEQ than in those expressing the cyt-AEQ probe (Figure 3, light and dark gray lines respectively). Indeed, given the steep Ca^2+ ^response curve, significant light emission is detected only when the aequorin pool (or a significant part of it) is exposed to high [Ca^2+^]. Accordingly, the probe for bulk cytosolic [Ca^2+^] shows a very small light emission upon the large Ca^2+ ^rise following Ca^2+ ^readdition to cell maintained in Ca^2+^-free media (i.e. upon capacitative Ca^2+ ^entry). Figures 3A and 3B illustrate also the results obtained with the cells co-transfected with the βarr2-AEQ and β_2_AR cDNAs, treated or not with isoprenaline (40 μM) for 1 min prior to Ca^2+ ^re-addition. In the untreated cells the increase of light emission upon Ca^2+ ^re-addition was only slightly higher than that observed in the cells transfected with cyt-AEQ (Figure 3A, black line), whereas in those treated with the β_2_AR agonist it was much higher, almost as high as that observed with the plasma membrane addressed SNAP-AEQ probe (Figure 3B, black line). Similar results were obtained in the cells transfected with the PKCβII-AEQ cDNA and then tested according to the same protocol, with the exception that the PKC stimulant, PMA (1 μM), was administered for 5 min before Ca^2+ ^re-addition (black traces). Also in this case the emission peak of the stimulated cells was nearly 10-fold higher than that of resting cells, reaching levels analogous to those of the SNAP-AEQ-transfected cells (compare the traces of Figure 3C and 3D).

### Automatization assay

**Figure 4:**
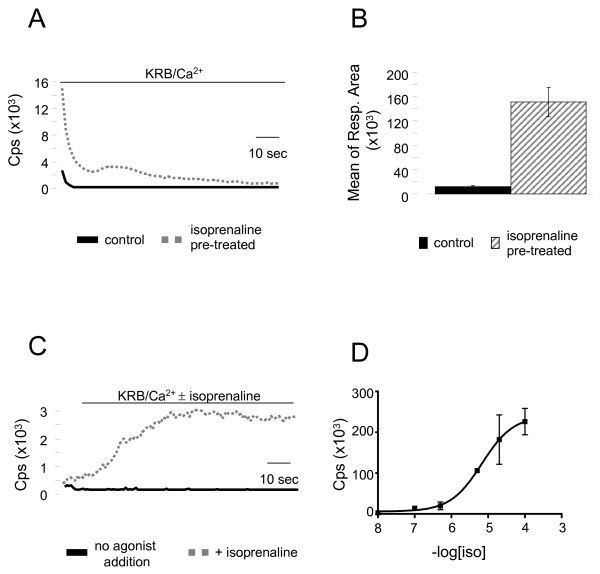
**Automated tracing of plasma membrane translocation aequorin responses**. **A**. The βarr2-AEQ probe was used to perform typical screening assays in an automated luminescence plate reader. Panel A shows the recording of the aequorin peaks elicited by Ca^2+ ^re-addition to the KRB bathing HeLa cells co-transfected with βarr2-AEQ and βAR. The cells were pre-treated (1 min) or not with isoprenaline (40 μM). The same data are represented in **(B) **as mean of integral response area (βarr2-AEQ control 11,909 ± 2,351 cps n = 15, βarr2-AEQ + isoprenaline 151,300 ± 24,090 cps n = 11). Panel **C **illustrates the time course of βarr2-AEQ probe translocation to the plasma membrane of HeLa cells co-transfected with βAR. The light responses were induced by addition (dotted line) or not (solid line) of isoprenaline 40 μM in KRB/Ca^2+^. **D**. Concentration response curve of cells co-transfected with βarr2-AEQ and βAR, pretreated with various concentrations of isoprenaline. The aequorin light emission responses were triggered by reintroduction of Ca^2+ ^as in Figure 3.

The power of the approach was further investigated by carrying out automated tracing assays in a luminescence plate reader, using 24-well plates governed by a robot. Figure 4 illustrates results obtained in βarr2-AEQ/β_2_AR cDNAs expressing HeLa cells stimulated with isoprenaline (40 μM). At first, we verified whether in this experimental setup, in which luminescence is not recorded in the injection phase and thus the upstroke of the rise is lost, a reproducible difference in aequorin light emission can be recorded when the βarr2-AEQ chimera translocates to the plasma membrane. Figures 4A and 4B show that this is indeed the case. The recording of the aequorin peaks elicited by Ca^2+ ^re-addition to KRB in each of the multi-well plates was in fact much higher in the treated cells than in the controls. Additional parameters were investigated. Figure 4C shows the time-course of the βarr2-AEQ translocation to the plasma membrane upon agonist addition. The maximal response, observed after approx. 30 s of stimulation, was followed by a persistent plateau. The concentration dependence of the isoprenaline-induced translocation of βarr2-AEQ is illustrated in Figure 4D. Detectable increases were observed at concentrations as low as 10^-8 ^M and maximal responses at 10^-4 ^M. Interestingly, this concentration response curve matches closely the curve of βarr2 binding to the β_2_AR reported previously in CLC12 cells using the β-galactosidase complementation procedure [12].

## Discussion

The possibility of randomly developing thousands of new drugs by combinatorial chemistry, and to rapidly develop lead compounds, represents an immense value in pharmaceutical research. To take full advantage of this technological potential, however, screening methodologies must be available that are robust, reproducible and amenable to medium/high throughput applications. In some cases (see the analysis of Ca^2+ ^homeostasis), the tools commonly employed in basic research, the intracellularly trappable fluorescent dyes, such as fura-2 or Fluo-3, proved highly effective in high-throughput screenings, and are commonly employed for the development of the vast array of drugs acting on Ca^2+ ^signalling [13]. These probes, however, can monitor one of the earliest events following receptor activation, but the data thus obtained are difficult to interpret for the understanding of other downstream signalling processes: protein translocation to the plasma-membrane is a typical example: it is a signalling step common to many diverse pathways (from RTKs and GPCRs, to the activation of specific enzymatic effectors) [14-17], but the currently available probes and techniques do not allow wide-range screenings or require complex and error-prone data analyses.

In this contribution, we have developed a novel approach that utilizes a Ca^2+ ^sensitive photoprotein, aequorin, for a task different from the measurement of calcium signalling itself, and stems from an experimental observation and a technical aspect. The first is the measurement of the [Ca^2+^] in the thin rim of cytoplasm beneath the plasma membrane, where both at rest and in particular upon stimulation the flow of Ca^2+ ^from the extracellular medium produces a local [Ca^2+^] that is at least one order of magnitude higher than in the deeper, bulk cytosol [8]. Thus, aequorin can act as a tag of protein translocation, as the simple movement of the tagged protein to the plasma membrane markedly changes the [Ca^2+^] to which it is exposed. The second startpoint of the new methodology is an intrinsic property of the photoprotein, i.e. the logarithmic correlation between [Ca^2+^] and light emission [7]. This property, that greatly increases light output with relatively small [Ca^2+^] increases, represents in most cases an experimental concern, as it tends to amplify the contribution of highly responding cells or cell domains in signalling studies. In the case of the new methodology reported in this paper, the high reponsiveness of the photoprotein represents a major plus, as it allows to detect protein translocation (e.g. a cytosolic adapter of the plasma membrane receptor) also when only a fraction of the protein actually moves within the cell. Together with the other advantages of a luminescence-based probe (no background signal, for the lack of other luminescent molecules within the cell, the simple geometry of the detection apparatus, etc.), this property provides a striking advantage over existing methodologies (GFP-based translocation assays, immunoblotting of subcellular fractions, etc.).

We have thus developed aequorin-based probes, that allow to detect the translocation of two classes of signalling proteins, a receptor adapter (β-arrestin, recognizing all classes of GPCRs) [18] and an important enzyme transducer, a member of the broad and complex family of PKC [3]. The engineered probes, βarr2-AEQ and PKCβ-AEQ were uniformly distributed in the cytosol at rest, and translocated to the plasma membrane upon stimulation with suitable compounds (isoprenaline and PMA respectively), triggering a dramatic increase in aequorin photon emission. In both cases, the probes proved very effective also with automated multi-well detectors. For β-arrestin, the pharmacological characterization of a receptor agonist proved its reliability, not only in the identification of active compounds, but also in the complete assessment of its functional properties.

## Conclusions

Overall, the understanding of the subcellular heterogeneity of Ca^2+ ^homeostasis and the experience with a recombinant luminescent Ca^2+ ^probe has allowed us to develop a robust, highly reproducible methodology for monitoring, in research applications and screening platforms, the translocation of specific proteins to the plasma membrane. This innovation will allow deciphering, and pharmacologically tackling, a critical step in a wide variety of signalling pathways including the whole complement of GPCRs and RTKs and kinases recruited to the plasma membrane independently of receptor activation [16,17]. This cellular assay, based on a single reporter protein and the unique single-to-noise ratio of the luminescent probe, meets the expanding demand for cost-effective screening procedures. It may therefore prove a highly valuable tool for identifying new blockbuster drugs in compound libraries of thousands of chemicals.

## Methods

### Plasmids and sequences

The sequence coding for the bovine β-arrestin-2 protein was a gift of dr. V. Schiaffino (Milan, Italy). The cDNA was amplified with the following primers: forward, 5'-GGGTACCGCCACCATGGGGGAGAAACCC-3' (KpnI site underlined); reverse, 5'-GAAGCTTCATGCAGAACTGGTCGTC-3' (HindIII site underlined), and then subcloned in the pSCA vector (StrataClone™). The sequence was excised from the pSCA vector and inserted in frame upstream of the cDNA coding for aequorin protein into the pcDNA3 vector, previously cut KpnI-HindIII.

The sequence coding for the β_2 _adrenergic receptor was amplified with the following primers: forward, 5'-GCGGCCGCGCCACCATGGGGCAACCCGGGAA-3' (NotI site underlined); reverse, 5'-CCGGATTCCGGTTACAGCAGTGAGTCATTTGTAC-3' (BamHI site underlined), subcloned in the pSCA vector and finally cloned into the pcDNA3 vector previously cut EcoRI. cyt-AEQ [11], and aequorin targeted to the subplasmamembrane region, SNAP-AEQ [8] were generated as previously described.

The PKCβII-AEQ probe was constructed cloning upstream the aequorin cDNA in pcDNA3 vector, previously cut KpnI, the sequence coding for the PKCβII, excised KpnI from the chimera PKCβII -GFP, present in our laboratories [5].

### Cell culture and transfection

HeLa cells were grown in Dulbecco's modified Eagle's medium (DMEM), supplemented with 10% fetal bovine serum (FBS), 2 mM L-glutamine, 0.25 mg/l benzylpenicillin solution, 10 U.I./I streptomycin solution. For aequorin measurements 50,000 cells were seeded either in 24-well plate Falcon or onto 13 mm glass coverslips; for immunofluorescence detection 100,000 cells were seeded on 24 mm glass coverslips. At 50% confluence the cells were transfected using the calcium-phosphate technique:

- media was replaced on each well, 1 h before transfection, with 1 or 2 ml of fresh media (DMEM + 10% FBS + pen/strep)

- cells were transfected using 4 ug (13 mm or 24 well) or 8 ug (24 mm) of total DNA

- day after medium was replaced and experiments were performed 36 h after transfection.

### Immunofluorescence analysis

Cells, seeded onto 24 mm coverslips, were transiently transfected with a total amount of 8 μg of the appropriate plasmid DNAs (βarr2-AEQ and β_2_AR/empty vector in a 1:3 ratio or PKCβII-AEQ alone). 36 h after transfection cells were stimulated with isoprenaline (40 μM, 1 min) or PMA (1 μM, 5 min), washed twice in PBS and fixed for 20 min with 4% paraformaldehyde in PBS at room temperature, supplemented with quenching solution (0.1 M glycine in PBS) for 10 min, washed and permeabilized with 0.2% Triton X-100 for 20 min, rinsed three times with PBS and incubated for 90 min with 2% BSA in PBS to block nonspecific binding sites. The proteins of interest were identified with primary polyclonal rabbit anti-arrestin IgG (1:500, Calbiochem, UK) or a monoclonal mouse anti-PKCβII (1:200, Santa-Cruz Biotec., CA, USA) antibodies in PBS, 2% BSA, incubated overnight at 4°C in a wet chamber and revealed with the AlexaFluor 594 anti-rabbit and anti-mouse antibodies (Invitrogen, Molecular Probes, CA, USA), respectively, diluted at 1:1000 in PBS, 2% BSA. After washing, the cells were imaged with a Zeiss LSM 510 Confocal Laser Scanning Microscope (Carl Zeiss, Jena, Germany).

### Aequorin assay with single-well luminometer

Cells were transfected with a total amount of 4 μg of the appropriate plasmid DNAs (βarr2-AEQ/cyt-AEQ/SNAP-AEQ and β_2_AR in a 1:3 ratio or PKCβII-AEQ/cyt-AEQ/SNAP-AEQ alone). In order to potentiate Ca^2+ ^influx and thus Ca^2+ ^level just beneath the plasma membrane, 36 h after transfection cells were incubated for 1 h at 37°C, in KRB/EGTA (Krebs-Ringer modified buffer: NaCl 125 mM, KCl 5 mM, Na_3_PO_4 _1 mM, MgSO_4 _1 mM, glucose 5.5 mM, HEPES 20 mM, pH 7.4/EGTA 100 μM,) supplemented with 5 μM coelenterazine for aequorin reconstitution. Cells were then stimulated with isoprenaline 40 μM for 1 min or PMA 1 μM for 5 min and light emission was measured restoring Ca^2+ ^to the extracellular medium perfusing cells with KRB plus 1 mM CaCl_2_. To terminate the experiments and discharge the remaining aequorin, the medium was replaced with a water solution containing 100 μM digitonin and 10 mM CaCl_2_. All results are expressed as mean ± standard error (SE).

### Aequorin assay with automated luminometer

After the transfection and reconstitution procedures, isoprenaline 40 μM was added for 1 min before putting the multi-well plate in the instrument (MicrobetaJET, PerkinElmer, CA, USA). KRB supplemented with 2 mM CaCl_2 _was then injected and luminescence was recorded for 60 s. To terminate the experiments and discharge the remaining aequorin, a water solution containing 500 μM digitonin and 50 mM CaCl_2 _was injected.

To follow the kinetics of βarr2-AEQ translocation, isoprenaline 40 μM was added at the KRB/Ca^2+ ^injected. To generate the concentration response curve, cells were treated with increasing concentration of isoprenaline (0.1-100 μM). All results are expressed as mean ± standard error (SE).

## Authors' contributions

All authors contributed extensively to the work presented in this paper. CG, AR, CA, GS and LB performed experiments; RR, TP, MB, JM and PP analyzed data. The paper was written by CG, JM, RR and PP. All authors read and approved the final manuscript.
